# Can radial bone mineral density predict spinal bone mineral density in patients with advanced Duchenne muscular dystrophy?

**DOI:** 10.1097/MD.0000000000012303

**Published:** 2018-10-05

**Authors:** Eunyoung Kim, Han Eol Cho, Ji Ho Jung, Jang Woo Lee, Won Ah Choi, Seong-Woong Kang

**Affiliations:** a Department of Rehabilitation Medicine and Rehabilitation Institute of Neuromuscular Disease; b Pulmonary Rehabilitation Center, Gangnam Severance Hospital, Yonsei University College of Medicine, Seoul; c Department of Physical Medicine and Rehabilitation, National Health Insurance Service Ilsan Hospital, Goyang, Korea.

**Keywords:** bone mineral density, dual X-ray absorptiometry, Duchenne muscular dystrophy, osteoporosis

## Abstract

In advanced Duchenne muscular dystrophy (DMD), patients with high bone fracture risk due to osteoporosis, it is difficult to measure spinal bone mineral density (BMD) because of maintaining proper posture. This study began with the idea that if we diagnose and manage osteoporosis by predicting spinal BMD through easily testable radial BMD, we could prevent fracture and improve quality of life in DMD patients. In 61 DMD patients aged 20 years or older who were admitted to Gangnam Severance Hospital from April 2013 to May 2015, radial BMD and spinal BMD were measured to compare their Z-scores. In 45 patients, the z-score was less than −2.0 in spinal BMD defined as osteoporosis. And the optimal range of Z-score in the radius was −5.2 to −5.0 (sensitivity 78.9%, specificity 71.4%). Only through the radius BMD, spinal BMD can be predicted and we suggest appropriate times for treatments.

## 1 Introduction

Duchenne muscular dystrophy (DMD), an X-linked recessive disorder, is the most common progressive muscular dystrophy developed in about 1 in 5000 male births.^[1]^ Initial DMD symptoms include proximal leg weakness and muscle wasting that eventually leads to difficulties with physical activity. As muscular weakness progresses, they become nonambulatory and show highly sedentary behavior with cardiopulmonary complications and musculoskeletal deformity.

It is known that DMD patients are vulnerable to low bone mass and osteoporosis, which is the most common metabolic bone disease that eventually leads to an increase of fragility fractures. A study revealed that fractures arose in 20% of DMD patients, 65% of whom turned out to be wheelchair-dependent patients.^[2]^ It is reported that DMD patients have several major risk factors for poor bone health, including delayed appearance of ossification centers especially in long and flat bones, reduced weight-bearing exercise due to muscular weakness during the important period of growth, used long-term treatment with glucocorticoids and reduced mobility causing reduced exposure to sunlight.^[3–5]^

Bone densitometry also called dual-energy x-ray absorptiometry (DXA), has used as a major tool for osteoporosis risk assessment, while the optimal method and measurement site are still controversial.^[6]^ Bone density in the spine decreases first because the bone turnover of this trabecular bone is greater than that of other skeletal sites.^[7]^ However, it is often difficult to evaluate spinal bone mineral density (BMD) in advanced DMD patients because they are not able to pose properly due to contracture of lower extremities and severe scoliosis, or metallic instruments of spine after scoliosis correction. For those patients, it is easier to examine radial BMD. But until now, no widely accepted values to evaluate osteoporosis via radial BMD have been developed.

Assessments of BMD by DXA are expressed as 2 measures, T-score and Z-score. T-score indicates standard deviation for healthy 30-year-old subjects, and Z-score for age-matched population. In World Health Organization (WHO), osteoporosis was defined with T-score on the basis of fracture risk in postmenopausal Caucasian women. Therefore, the correlation between fracture risk and BMD is not clear in pre-menopausal women and men aged 50 or less,^[8]^ in addition, T-score application is not appropriate in children aged 20 or less who have not yet reached peak bone mass.^[9]^ Accordingly, International Society for Clinical Densitometry defines the diagnosis of osteoporosis as a BMD Z-score of ≤ −2.0 in pre-menopausal women, men aged 50 or less, and children (male or female aged 20 or less).^[10]^

The purpose of the study is to investigate the correlation between radial and spinal BMD and the possibility of predicting spinal BMD by performing a radial BMD assessment in patients with advanced non-ambulatory DMD.

## 2 Materials and methods

As a retrospective study, we analyzed data collected from Department of Rehabilitation Medicine in Gangnam Severance Hospital from April 2013 to May 2015. We included only those patients in whom the diagnosis of DMD was confirmed by muscle biopsy or genetic tests. All of them were advanced DMD patients aged 20 or more using a ventilator, showing entire loss of ambulatory function. Subjects had no previous history of long-term therapy with glucocorticoids. Those who were unable to be evaluated BMD of lumbar vertebrae L1 to L4 due to scoliosis operation or abnormal postures, and who had a history of vertebral fracture, were excluded. Only subjects who meet these conditions submitted a consent form before the examination in compliance with ethical approval.

BMD was measured with DXA at the radius and lumbar (L1-L4) spine. BMD was expressed as an absolute value (mg/cm^2^) and Z-score. The Z-scores were calculated with reference to BMD (similarly adjusted for body surface and vertebral volume) of a population of age-matched healthy people.

### 2.1 Statistical analysis

Data analysis was performed by a software (SPSS, version 23.0 (IBM Corp. 2015)). For a measure of the strength of the linear relationship between the radial and spinal Z-score, Pearson's correlation was employed. The sensitivity and specificity of the radial Z-score as indicators of the spinal Z-score were determined with cut-off values. Receiver operating characteristic (ROC) curves and area under the curve (AUC) for ROCs were obtained by plotting sensitivity against the false-positive rate (1−specificity). The Youden's index (J, sensitivity + specificity − 1) was used to determine optimal cut-off values of the radial Z-score for identification of the spinal Z-score under −2.0.

## 3 Results

**Figure 1:**
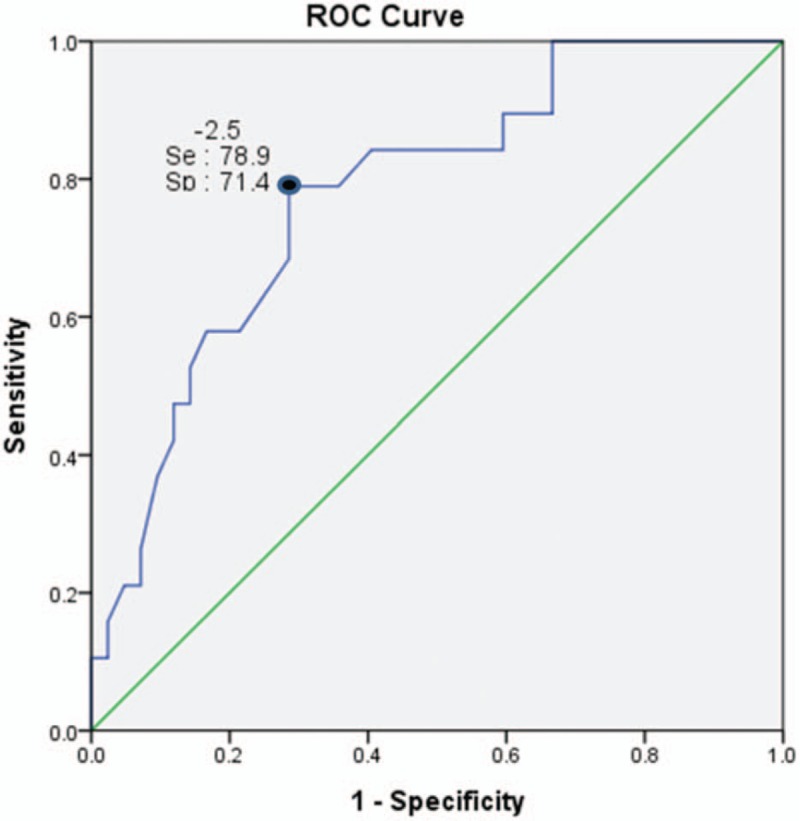
ROC for the prediction of the Z-score in the spine using Z-score in the radius. ROC = receiver operating characteristic curve.

A total of 61 subjects at a mean age of 27.2 ± 5.6 years were included (Table 1) in the study. The spinal Z-scores of 45 patients (73.7%) were −2.0 or lower. The Z-score of the spine and radius were correlated well with one another (r = 0.599, *P* < .001). Figure 1 shows the ROC curves for the prediction of osteoporosis. The area under ROC curve for the prediction of the Z-score in the spine under −2.0 was found to be 0.781 (*P* = .001) in the overall data. The optimal Z-score in the radius was in the range of −5.2 to −5.0 (sensitivity 78.9%, specificity 71.4%).

**Table 1:**
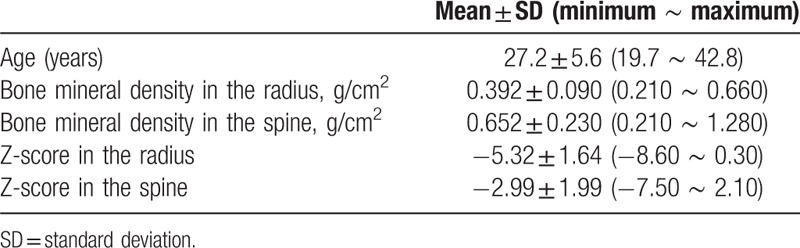
General characteristics of participants.

## 4 Discussion

According to the WHO criteria, osteoporosis is defined based on the evaluation of BMD, and DXA is an assessment tool most commonly employed. It is diagnosed using T-score and Z-score by assessing BMD in the hip, spine, and forearm, in particular, the intervention time can be determined by evaluating the high risk-group for osteoporosis through a fracture history in the hip or spine.^[11]^

However, the correlation between the T-score and the Z-score has not yet been established in each site including the hip, spine, and forearm. In a study aimed at healthy postmenopausal women, BMD in the forearm was compared to that of the spine and femur by DXA, consequently demonstrating that it is difficult to determine whether osteoporosis is developed in the different sites using unique T-score.^[12]^ In another study, the strongest association between the distal radius and spine was respectively confirmed by Quantitative CT (QCT) in healthy premenopausal women, healthy postmenopausal women, and osteoporotic postmenopausal women. In the case of healthy women, the strongest capability for assessment of bone loss was found in trabecular bone of the distal radius and spine, on the contrary, the weakest association was observed in osteoporotic women. The results revealed that it is possible to distinguish between osteoporotic and nonosteoporotic patients and monitor those patients just by evaluating the distal radius.^[13]^ Given the 2 studies, it is difficult and still controversial to predict osteoporosis just by the evaluation of BMD in a certain part of the hip, spine, and especially forearm. In particular, the correlation between BMD of the radius and that of the lumbar and femur in DMD patients as this study has not been investigated, and the comparison and analysis study for Z-score by DXA was also unsatisfactory.

This study was the first to show sensitivity and specificity of predicting BMD of vertebrae as a Z-score of radius evaluated by DXA. In a study comparing BMD of distal radius and spine or femoral neck in normal healthy adults, sensitivity and specificity for right radius were 90.00% to 95.45% and 53.85% to 73.68%, respectively; and for left radius, sensitivity and specificity were 85.00% to 96.67% and 57.69% to 81.58%, respectively.^[14]^ In addition, there was a study comparing BMD of heel evaluated by DXA with laser (DXL) to BMD of spine, hip, and total body assessed by DXA in children under 20 years of age, including those with muscular dystrophy. In the study, sensitivity was 90%, and specificity was 86%, 92%, and 95% for total body, spine, and hip, respectively.^[15]^ In our study, sensitivity was 78.9%, and specificity was 71.4%. Results in our study were not worse compared to those of previous studies, although variables and assessment tools were different.

It is considered that the evaluation of BMD for the radius contributes to the diagnosis of osteoporosis, because the problems such as overestimation of BMD by vertebral fracture, overlying metal through surgical procedure, and poor positioning due to severe scoliosis, and underestimation of BMD by low body weight of general DMD child patients, can be excluded. If patients with DMD could not check spinal BMD for some reasons, radial BMD may be used to predict spinal BMD. Moreover, as current medications for osteoporosis are covered by national health insurance only based on T- and Z-score of BMD for central bones including the spine and femur, the examination performed only in the radial bone has still a limitation in terms of insurance coverage. However, it is believed that this study is capable of suggesting another standard to the insurance system of Korea.

Another strength of the current study is that this is the first investigation targeting steroid-naive patients, and it also evaluates the natural history of BMD in DMD patients.

This study shows a limitation in that the evaluation was carried out not including calcium and vitamin D intake status, nutritive conditions such as food intake, degree of exposure to sunlight, and sitting endurance. In addition, patients aged less than 20 were excluded in the study because not covered by health insurance depending on the basis of Korean National Health Insurance (all people should obligatorily have the insurance). Particularly, this study employed DXA to assess the areal BMD, which had, however, a weak point to underestimate the true density value for smaller bones and overestimate it for larger bones.^[16]^ Therefore, it is also considered a limitation that QCT was not introduced as a tool capable of assessing not only BMD but also bone mineral content (BMC).

It is crucial to diagnose and treat osteoporosis in the proper period in DMD child patients vulnerable to poor bone health from an endocrine, nutritional, and behavioral viewpoint in preventing fractures. Recently, the safety of bisphosphonate has been demonstrated in pediatric osteoporosis, although the effect of its long-term use is under constant research, and treatment with appropriate drugs is important at the right time.^[17]^ However, this point has not been considered until now in DMD child patients in which BMD for the lumbar spine and femur is hard to be assessed. This study indicated that the intervention can be performed in the appropriate period by considering the Z-score of the spine just by evaluation for the radius. It is possible to prevent fractures by figuring out the correct time point of management through the correlation between Z-score of the radius and vertebral fracture. Currently, the correct estimation of a fracture risk with a Z-score has not been established, thus a study for this should be conducted in the future.

## 5 Conclusion

In this study, we propose a novel standard to diagnose and treat osteoporosis of central bones simply by evaluating the radius based on the correlation between radial and vertebral bone mineral densities.

## Author contributions

**Conceptualization:** Eunyoung Kim, Han Eol Cho.

**Data curation:** Han Eol Cho, Jiho Jung.

**Supervision:** Seong-Woong Kang, Won Ah Choi, Jang Woo Lee.

**Writing – original draft:** Eunyoung Kim, Han Eol Cho.

**Writing – review & editing:** Eunyoung Kim, Seong-Woong Kang.

Seong-Woong Kang orcid: 0000-0002-7279-3893
